# F167. ACCESS, UNDERSTAND, APPRAISE AND APPLY TO / OF HEALTH INFORMATION AND HEALTH LITERACY IN INDIVIDUALS AT-RISK FOR PSYCHOSIS: A SYSTEMATIC REVIEW

**DOI:** 10.1093/schbul/sby017.698

**Published:** 2018-04-01

**Authors:** Mauro Seves, Theresa Haidl, Susanne Eggers, Ayda Rostamzadeh, Anna Genske, Saskia Jünger, Christiane Woopen, Frank Jessen, Stephan Ruhrmann

**Affiliations:** 1 University of Cologne; 2 Institute for the History of Medicine and Medical Ethics, University of Cologne

## Abstract

**Background:**

Numerous studies suggest that health literacy (HL) plays a crucial role in maintaining and improving individual health. Furthermore, empirical findings highlight the relation between levels of a person’s HL and clinical outcomes. So far, there are no reviews, which investigate HL in individuals at-risk for psychosis. The aim of the current review is to assess how individuals at risk of developing a first episode of psychosis gain access to, understand, evaluate and apply risk-related health information.

**Methods:**

A mixed-methods approach was used to analyze and synthesize a variety of study types including qualitative and quantitative studies. Search strategy, screening and data selection have been carried out according to the PRISMA criteria. The systematic search was applied on peer-reviewed literature in PUBMED, Cochrane Library, PsycINFO and Web of Science. Studies were included if participants met clinical high risk criteria (CHR), including the basic symptom criterion (BS) and the ultra-high risk (UHR) criteria. The UHR criteria comprise the attenuated psychotic symptom criterion (APS), the brief limited psychotic symptom criterion (BLIPS) and the genetic risk and functional decline criterion (GRDP) Furthermore, studies must have used validated HL measures or any operationalization of the HL’s subdimensions (access, understanding, appraisal, decision-making or action) as a primary outcome. A third inclusion criterion comprised that the concept of HL or one of the four dimensions was mentioned in title or abstract. Data extraction and synthesis was implemented according to existing recommendations for appraising evidence from different study types. The quality of the included studies was evaluated and related to the study results.

**Results:**

The search string returned 10587 papers. After data extraction 15 quantitative as well as 4 qualitative studies and 3 reviews were included. The Quality assessment evaluated 12 publications as “good”, 9 as “fair” and one paper as “poor”. Only one of the studies assessed HL with as primary outcome. In the other studies, the five different subdimensions of HL were investigated as a secondary outcome respectively mentioned in the paper. “Gaining Access” was examined in 18 of the 22 studies. “Understanding” has been assessed in 7 publications. “Appraise” was examined in 9 studies. “Apply decision making” and “Apply health behavior” were investigated in 1 of 8 studies. Since none of the included publications operationalized neither HL nor the subdimensions of HL with a validated measure, no explicit influencing factors could be found.

**Discussion:**

Quantitative and qualitative evidence indicates that subjects at-risk for psychosis describe a lack of understanding about their state and fear stigmatization that might lead to dysfunctional coping strategies, such as ignoring and hiding symptoms. Affected subjects are eager to be informed about their condition and describe favoured channels for obtaining information. The internet, family members, school personnel and GP’s play a crucial role in gain access to, understand, evaluate and apply risk-related health information. The results clearly highlight that more research should be dedicated to HL in individuals at risk of developing a psychosis. Further studies should explore the relation between HL and clinical outcomes in this target population by assessing the underlining constructs with validated tools.

